# Subregional Density of Neurons, Neurofibrillary Tangles and Amyloid Plaques in the Hippocampus of Patients With Alzheimer’s Disease

**DOI:** 10.3389/fnana.2019.00099

**Published:** 2019-12-19

**Authors:** Diana Furcila, Marta Domínguez-Álvaro, Javier DeFelipe, Lidia Alonso-Nanclares

**Affiliations:** ^1^Cajal Laboratory of Cortical Circuits, Centre for Biomedical Technology (CTB), Universidad Politécnica de Madrid, Madrid, Spain; ^2^Network Biomedical Research Center on Neurodegenerative Diseases (CIBERNED), Madrid, Spain; ^3^Cajal Institute (CSIC), Madrid, Spain

**Keywords:** amyloid-β protein, dementia, hippocampal formation, neuronal density, plaques density, neurodegeneration, stereology

## Abstract

A variety of anatomical alterations have been reported in the hippocampal formation of patients with Alzheimer’s Disease (AD) and these alterations have been correlated with cognitive symptoms in the early stages of the disease. Major hallmarks in AD are the presence of paired helical filaments of tau protein (PHF_Tau_) within neurons, also known as neurofibrillary tangles (NFTs), and aggregates of amyloid-β protein (Aβ) which form plaques in the extracellular space. Nevertheless, how the density of plaques and NFTs relate to the severity of cell loss and cognitive decline is not yet clear. The aim of the present study was to further examine the possible relationship of both Aβ plaques and NFTs with neuronal loss in several hippocampal fields (DG, CA3, CA1, and subiculum) of 11 demented AD patients. For this purpose, using stereological techniques, we compared neuronal densities (Nissl-stained, and immunoreactive neurons for NeuN) with: (i) numbers of neurons immunostained for two isoforms of PHF_Tau_ (PHF_Tau-AT8_ and PHF_Tau-pS396_); and (ii) number of Aβ plaques. We found that CA1 showed the highest number of NFTs and Aβ plaques, whereas DG and CA3 displayed the lowest number of these markers. Furthermore, AD patients showed a variable neuronal loss in CA1 due to tangle-related cell death, which seems to correlate with the presence of extracellular tangles.

## Introduction

Major hallmarks in Alzheimer’s disease (AD) are the presence of paired helical filaments of tau protein (PHF_Tau_) within neurons, also known as neurofibrillary tangles (NFTs), and aggregates of amyloid-β protein (Aβ) which form plaques in the extracellular space (Grundke-Iqbal et al., 1986; Goedert et al., 1988; Avila, 2004; Hyman et al., 2012; Takahashi et al., 2017). As previously discussed in Garcia-Marin et al. (2009), plaques and NFTs are mostly found in the cerebral cortex (entorhinal cortex, hippocampal formation and neocortex), where both their number and the proportion of the cortex affected increases progressively as the disease advances (Braak and Braak, 1991; Dickson, 1997; Thal et al., 2002). These pathological changes have also been found in subcortical structures such as the amygdala, nucleus basalis, thalamus, locus coeruleus, and raphe nuclei, particularly during the late stages of AD (e.g., Esiri et al., 1997). Accordingly, multiple neuronal circuits and neurotransmitter systems may be altered in the brain of AD patients.

It has been described that these two proteins (Aβ and PHF_Tau_) act together and even enhance each other (DeVos et al., 2017; Polanco et al., 2017; Furcila et al., 2018), but it is still unknown which protein triggers degeneration. Their accumulation in the cerebral cortex starts at different regions; in initial stages of AD, PHF_Tau_ is seen in the medial temporal lobe (MTL; Braak and Braak, 1991), and Aβ protein starts to form aggregates in neocortical regions, particularly in temporal and frontal lobe (Thal et al., 2002; Sepulcre et al., 2013). The main controversy is still whether it is Aβ, PHF_Tau_ or their interaction that correlates with the disease progression in AD (Nelson et al., 2012; Bloom, 2014). In initial stages of the disease, a number of alterations, such as neuronal loss and changes in the pyramidal cell morphology, among others, have been reported in hippocampal regions from AD patients (Hyman et al., 1990; West et al., 2004; Merino-Serrais et al., 2011; Andrade-Moraes et al., 2013; Llorens-Martín et al., 2014). AD cognitive symptoms in early stages are loss of context, disorientation and autobiographical memory impairment (Dubois et al., 2007; Petersen et al., 2009). The MTL and, in particular, the hippocampal formation and adjacent cortex support these functions (Hyman et al., 1986; Amaral and Lavenex, 2007). It is well known that a variety of changes occur in the hippocampal regions of AD patients, such as a decrease in the number of neurons, as well as neuronal alterations (for example; changes in the morphology of the dendritic arbor and spines); the presence of neuropil threads and Aβ plaques; and microvascular changes (e.g., Hyman et al., 1990; West et al., 2004; von Gunten et al., 2006; Bouras et al., 2006; Giannakopoulos et al., 2003; Andrade-Moraes et al., 2013; Llorens-Martín et al., 2014). It has been proposed that the presence of PHF_Tau_ aggregates and Aβ-ir plaques related to AD may represent a toxic environment for hippocampal neurons (Ittner and Götz, 2011). However, it has been demonstrated that CA1 neurons show a heterogeneous expression pattern of PHF_Tau_ proteins. For example, in a previous study performed in our laboratory in CA1 regarding the co-expression of PHF_Tau-pS396_ and PHF_Tau-AT8_, we found that most (64%) of the labeled neurons expressed only PHF_Tau-pS396_, whereas 28% displayed both markers and 8% showed only PHF_Tau-AT8-ir_ (Furcila et al., 2018).

The aim of the present study was to further examine the relationship of both proteins in AD and possible neuronal loss in different hippocampal regions. For this purpose, we determined possible differences between regions, markers and cases, by comparing neuronal densities (Nissl-stained, NeuN-ir, PHF_Tau-AT8-ir_ and PHF_Tau-pS396-ir_ neurons) and Aβ-ir plaques.

To label PHF_Tau-ir_ neurons, we chose the antibody PHF_Tau-AT8_ because it is commonly used to classify the neurofibrillary degeneration into stages known as the Braak stages (Braak et al., 2006), and the antibody PHF_Tau-pS396_ because phosphorylation at site S396 is commonly related to late stages of AD (Regalado-Reyes et al., 2019) and it is mostly found in NFTs (Kimura et al., 1996). For this purpose, we used stereological techniques for neurons, PHF_Tau_ and Aβ plaques stainings (Nissl; NeuN; Aβ; PHF_Tau-AT8_; PHF_Tau-pS396_) to study the DG, CA3, CA1 and subiculum from the hippocampus of 11 demented AD patients.

## Materials and Methods

### Tissue Preparation

Samples of human brain tissue from AD patients were obtained from 11 cases (post-mortem delay between 2:00 and 5:30 h; aged between 69 and 89 years upon death; Table 1) diagnosed with AD according to neuropathological examination as stated by Braak and Braak (1991) and CERAD (Mirra et al., 1991; Fillenbaum et al., 2008). These samples were obtained from two sources: Banc de Teixits Neurologics from Hospital Universitari Clinic de Barcelona (Spain) and Banco de Tejidos Fundación CIEN (Madrid, Spain), following the guidelines of the Helsinki Declaration and with the approval of the local Ethical Committees. Permission to publish the data about age and other demographic information was obtained from the patients or their next of kin along with the informed consent for use of the donor’s biological samples for research, according to national regulation on biobanks. We used coronal sections of the human hippocampus at the level of the hippocampal body (Mai et al., 2016). These tissue samples have been previously used in our laboratory (Furcila et al., 2018; Regalado-Reyes et al., 2019; case codes Az1–10 correspond to BCN1–10, and Az11 to VK26). Tissue blocks containing the hippocampal formation were fixed in a solution of 4% paraformaldehyde (for 24 h at 4°C), cryoprotected (30% sucrose solution for at least 24 h) and deep-frozen (−80°C). Blocks containing the hippocampus were cut with a sliding microtome at −40°C (Thermo Fischer Scientific, Waltham, MA, USA, MICROM, HM450; freeze unit, KS34, Massachusetts, USA), in serial coronal sections (50 μm thick) and collected in 0.1 M PB for further processing.

**Table 1:** Summary of clinical and pathological data.

Patient	Gender	Age at disease	Braak and CERAD Scales	AD progression (years)	Postmortem delay (h:m)
Az1	M	80	VI, C	10	4:30
Az2	F	70	V, C	12	2:00
Az3	F	79	V, C	2	5:30
Az4*	F	78	IV, B	9	5:30
Az5	F	69	V/VI, C	14	4:00
Az6*	F	69	V, C	7	5:00
Az7*	F	89	VI, C	-	4:15
Az8*	F	77	VI, C	8	5:45
Az9*	F	76	VI, C	8	5:00
Az10	F	71	V, C	9	5:00
Az11*	F	82	V, -	13	4:00

Braak Stages (Braak and Braak, 1991): I–II (NFTs in entorhinal cortex and closely related areas); III–IV (NFTs abundant in amygdala and hippocampus and extending slightly into the association cortex); V–VI (NFTs widely distributed throughout the neocortex and ultimately involving primary motor and sensory areas). CERAD Stages (Mirra et al., 1991; Fillenbaum et al., 2008): A (low density of neuritic plaques); B (intermediate density of neuritic plaques); C (high density of neuritic plaques). Code Az1-Az10 corresponds, respectively, to the codes BCN1-BCN10 used in previous articles from our laboratory, while Az11 corresponds to the code VK26. F, female; h, hours; *hippocampal sclerosis; m, minutes; M, male. (-) Data not available.

### Immunohistochemistry

**Figure 1:**
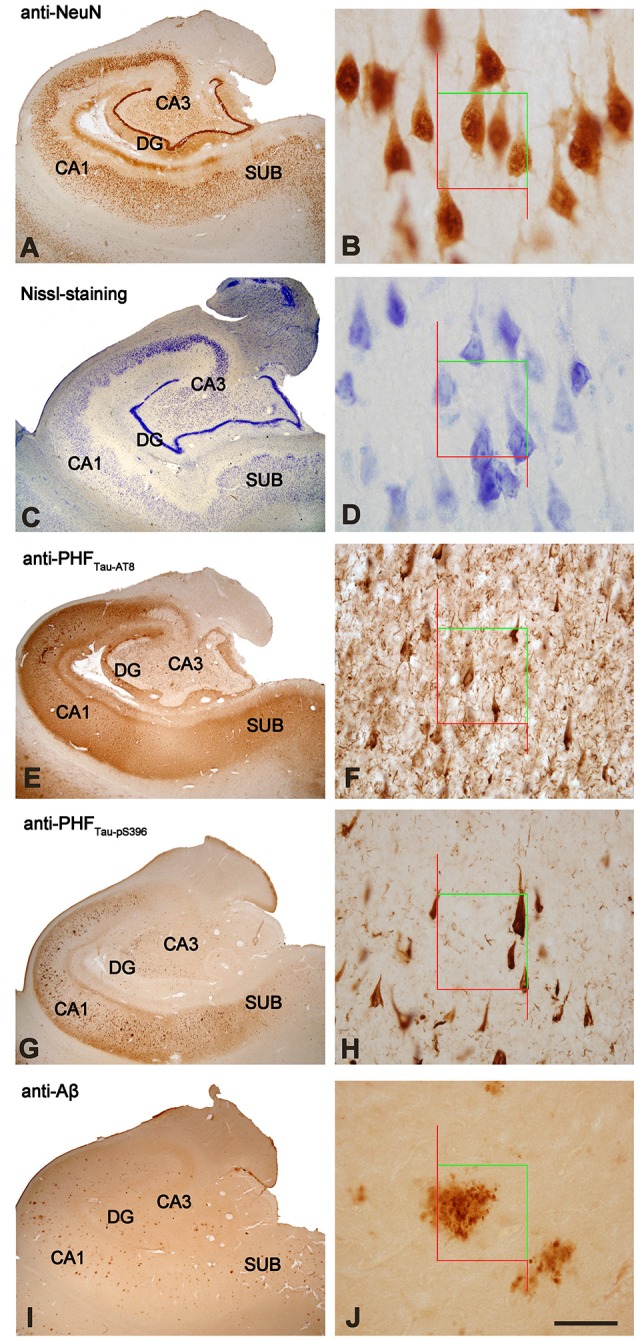
Stained hippocampal formation sections from an AD patient. Left column, low-power microphotographs of the hippocampal formation showing NeuN_-ir_ **(A)**, Nissl-staining **(C)**, PHF_Tau-AT8-ir_ **(E)**, PHF_Tau-pS396-ir_ **(G)** and Aβ_-ir_ **(I)** sections. Right column **(B,D,F,H,J)**, higher magnification of CA1 region to illustrate the applied counting frame (green lines correspond to the inclusion border and red lines to the exclusion border) used in the stereological procedure. Scale bar, shown in **(J)**, indicates 1,000 μm in **(A,C,E,G**,**I)**; 37 μm in **(B,D)**; 90 μm in **(F,H,J)**. DG: dentate gyrus; CA1-CA3: *cornu ammonis* fields; SUB: subiculum.

The sections selected from the hippocampal formation were processed for standard immunocytochemical techniques (Figure 1). Briefly, the sections were first treated to eliminate endogenous peroxidase (1% H_2_O_2_). Those sections selected for the staining for anti-Aβ were first treated with 88% formic acid (Sigma–Aldrich, No. 251364, St. Louis, MO, USA). The sections were then treated with 0.25% Triton-X in PB 0.1 M, 3% horse serum (NHS, Vector Laboratories Inc., Burlingame, CA, USA) and incubated overnight at 4°C with a specific primary antibody. On the following day, the sections were rinsed and processed with a biotinylated secondary antibody (horse anti-mouse or goat anti-rabbit IgG, 1:200, Vector Laboratories, Burlingame, CA, USA), and then with Avidin-Biotin Complex (Vectastain ABC Kit, Vector Laboratories Inc., Burlingame, CA, USA). Staining was visualized with the chromogen 3, 3′-diaminobenzidine (DAB: Sigma Aldrich, St. Louis, MO, USA). All sections were incubated until they turned light brown, with the same duration of incubation for all slides; the immunostaining was checked via light microscope before stopping the reaction in all slides. Then, the sections were incubated in 0.02% OsO4 in PB for 15 s at room temperature. Finally, the sections were mounted, dehydrated, cleared with Xilol and covered-slipped (DePeX; Merck KGaA 100579, 64271, Germany). The following primary antibodies were used: mouse anti-NeuN monoclonal antibody (1:2,000; Chemicon; MAB377, Temecula, CA, USA); mouse anti-PHF_Tau-AT8_ monoclonal antibody (1:2,000, MN1020, Thermo Scientific, Waltham, MA, USA); rabbit anti-PHF_Tau-pS396_ polyclonal antibody (1:2,000, Invitrogen, 44752G, CA, EEUU); and mouse anti-Aβ monoclonal antibody (clone 6F/3D, 1:50, Dako M0872, Glostrup, Denmark). Adjacent sections were Nissl-stained with toluidine blue (Figure 1).

To generate the figures, images were captured with a digital camera (Olympus DP70) attached to an Olympus BX51 light microscope (Olympus, Ballerup, Denmark) and Adobe Photoshop CS4 Extended 11.0.2 software (Adobe Systems, San Jose, CA, USA) was used to produce the figure plates.

### Estimation of Neuronal Densities

Densities of labeled neurons were estimated using a stereological method known as optical dissectors (Figure 1; West and Gundersen, 1990) with the aid of Stereo Investigator software (Stereo Investigator 11.0, MicroBright Field Inc., Williston, VT, USA), using its Optical Fractionator tool. Neuronal densities, expressed as the number of labeled neurons per volume, were estimated in CA3, CA1, and subiculum, using Nissl-stained sections and NeuN-, PHF_Tau-AT8-_ and PHF_Tau-pS396_-immunostained sections. Nissl-stained and NeuN-immunostained sections were used to identify the boundaries within the hippocampus.

After randomly selecting a starting point, six sections were chosen at equally spaced intervals. Optical dissectors were made with an oil immersion ×100 objective for both the NeuN-immunostained and Nissl-stained sections, on an average surface of 2,050 μm^2^. The depth of the optical dissectors was 10 μm, rendering a study volume of 20,500 μm^3^ per optical dissector. An ×40 objective was used for the PHF_Tau_-immunostained sections, on a surface of 14,450 μm^2^. The depth of the optical dissectors in this case was also 10 μm, rendering a study volume of 144,500 μm^3^. Stereological parameters for each sample and neuronal marker were chosen. Since most neurons are located in the pyramidal cell layer, neuronal densities were estimated in this layer in the CA subfields and subiculum. In Nissl-stained sections, a neuron was only counted if the nucleolus was clearly identified in the optical plane along the vertical z-axis (Figure 1).

### Estimations of Amyloid Plaque Density and Volume

The number of Aβ_-ir_ plaques per volume was also estimated by the Optical Fractionator tool (Stereo Investigator) in DG, CA3, CA1 and subiculum (Figure 1). A minimum of six sections were selected for each patient, with equal intervals with an ×40 objective on a surface of 22,500 μm^2^ and with a dissector depth of 10 μm, rendering a study volume of 225,000 μm^3^ per optical dissector.

To estimate the Aβ-ir plaque volume, the edges of the plaque were delineated with the Nucleator tool with the aid of Stereo Investigator software (Gundersen, 1988). This tool provides the volume of each Aβ-ir plaque analyzed, as well as the relative volume occupied by them in each examined hippocampal subfield (Figure 1) to provide the percentage of tissue (%) occupied by Aβ-ir plaques.

### Tissue Shrinkage Estimation

Tissue shrinkage due to staining protocols was estimated measuring the section area and thickness before and after processing to correct the final values using Stereo Investigator software. The area of the section after processing was divided by the area value measured before processing, to obtain a shrinkage factor for any area measurement. The thickness was measured at 10 random points to estimate shrinkage along the z-axis (i.e., section compression). As a result, the brain tissue was estimated to have shrunk 30% in volume when processed for DAB and Nissl-staining: for the DAB-immunostaining, the average thickness of the unstained sections was 50.2 μm, and after immunostaining processing, it was 16.49 μm in NeuN-, PHF_Tau-_ and Aβ-immunostained sections; in Nissl-stained sections, the average thickness after processing was 17.4 μm. Thus, the final values of all stereological estimations were corrected to obtain an estimation of the pre-processing values.

### Statistical Analysis

To determine possible differences between regions, markers, and cases, statistical comparisons of neuronal densities (Nissl-stained, NeuN-ir, PHF_Tau-AT8-ir_, and PHFTau-pS396-ir neurons) and Aβ_-ir_ plaque data were carried out. When the data were parametric (normality and homoscedasticity criteria were met), we performed a *t*-student test and ANOVA test, followed by Bonferroni test for pair-wise comparisons. Normality and homoscedasticity were analyzed with the Shapiro–Wilk and Levene tests, respectively. When normality and homoscedasticity were not met, we used unpaired Mann–Whitney (MW) nonparametric *U*-test and Kruskal–Wallis test (KW), followed by the Mann–Whitney test (MW) for pair-wise comparisons. Correlation analysis was used to find possible relations between variables. Statistical procedures and graphs were carried out with GraphPad Prism 7 statistical package (Prism, San Diego, CA, USA) and SPSS program (IBM SPSS Statistics v25, IBM Corporation, Foster City, CA, USA).

## Results

In this study, divisions of the hippocampal fields were delineated on the basis of previous descriptions (Insausti and Amaral, 2012) and nomenclature for each region was previously used in Alonso-Nanclares et al. (2011). For the purposes of the present work, we will use the terms: DG, the hippocampus proper (subdivided into CA1, CA2 and CA3 fields) and the subiculum (Figure 1).

### Neuronal Density Estimations

**Figure 2:**
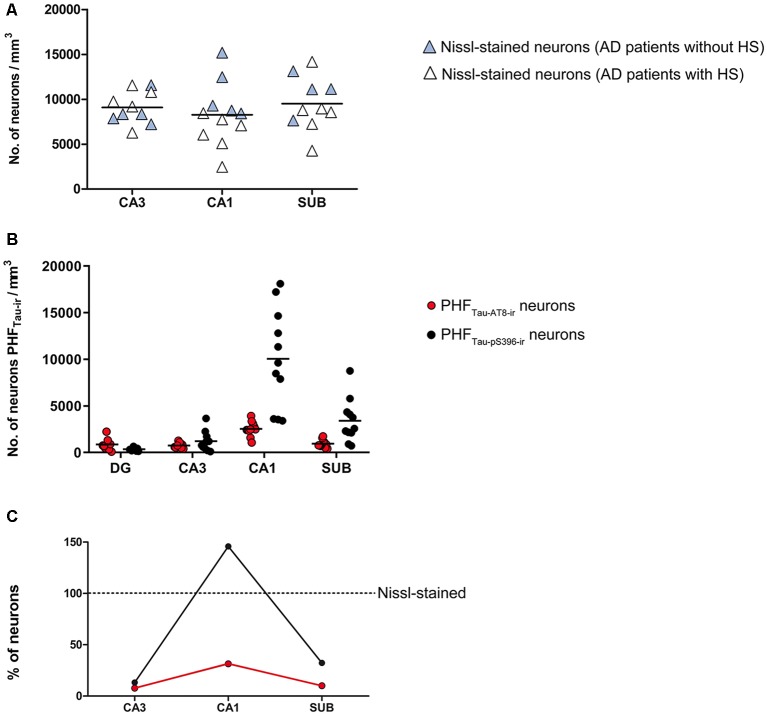
**(A)** Plot showing neuronal densities in examined hippocampal areas, estimated in Nissl-stained sections. The densities represented correspond to the number of labeled neurons per volume (mm^3^) for each analyzed case. White symbols correspond to the AD patients with HS. **(B)** Graph showing neuronal densities per volume of PHF_Tau-AT8-ir_ and PHF_Tau-pS396-ir_ neurons in the hippocampal fields analyzed, in AD patients. The densities represented correspond to the number of labeled neurons per volume (mm^3^) for each analyzed case.** (C)** Graphical representation of the percentages of PHF_Tau-AT8-ir_ and PHF_Tau-pS396-ir_ neurons, considering Nissl-stained neurons as the total neuronal population. Data correspond to the average percentages per region. Note that the percentage of PHF_Tau-pS396-ir_ elements surpasses the total neuronal population, which may be a consequence of extracellular labeled NFTs or “ghost tangles” (see text for further details). HS, Hippocampal sclerosis; DG, dentate gyrus; CA1–CA3, *cornu ammonis* fields; SUB, subiculum.

The overall neuronal density was estimated in both Nissl-stained and NeuN-immunostained sections in the pyramidal layer of CA3, CA1 and subiculum (Supplementary Table S1; Figure 2A). Other hippocampal regions were not used for neuronal counting due to technical difficulties, such as the dense packing of the granular cells in the DG and the reduced volume of CA2, which produce high error coefficients that are not acceptable when obtaining accurate estimations. No differences were observed in the number of neurons per volume when comparing both markers across regions (*T*-test: *p* = 0.13 in CA3; *p* = 0.73 in CA1 and *p* = 0.41 in Subiculum). Neuronal densities in AD patients showed a wide range of values in all analyzed regions (Figure 2A). Moreover, estimations performed in NeuN_-ir_ sections displayed a higher variability than those densities obtained in Nissl-stained sections (Supplementary Table S1). Unless otherwise specified, we will refer to the data obtained in Nissl estimations for the neuronal density.

**Figure 3:**
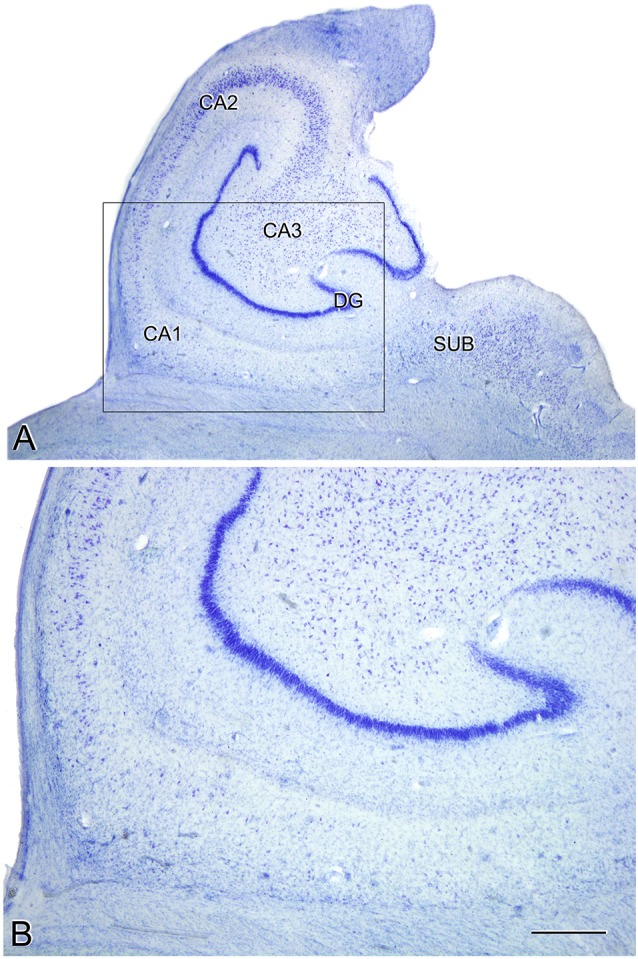
Microphotographs of the hippocampal formation from a Nissl-stained section of an AD patient (Az9) affected by hippocampal sclerosis **(A)**. Higher magnification of the boxed region in A shows the severe neuronal loss and gliosis in CA1 **(B)**. DG: dentate gyrus; CA1-CA3: *cornu ammonis* fields; SUB: subiculum. Scale bar (in **B**) indicates 2.25 mm in **(A)**, and 1,000 mm in **(B)**.

The estimated CA1 neuronal density was 8,852 ± 4,368 (mean ± SD; Supplementary Table S1). In six of the AD patients, we found a very low number of neurons in CA1, which was compatible with hippocampal sclerosis (HS), a pathological finding (Figure 3) which is often associated with AD (Dickson et al., 1994; Velez-Pardo et al., 2004; Attems and Jellinger, 2006; Amador-Ortiz et al., 2007). The neuronal densities found in CA1 from those AD patients with HS were 5,492 ± 2,029 neurons/mm^3^ (mean ± SD), whereas in AD patients without HS they were 12,884 ± 2,304 (Figure 2A). The latter values were similar to values found in CA1 from control cases (12,040 ± 1,746; see “Discussion” section). No statistical differences were observed in the number of neurons per volume (mm^3^) across fields (CA1, CA3, and subiculum) compared to each other (one way ANOVA; *p* = 0.6041; *F* = 0.5132).

### PHF_Tau-ir_ Studies

To examine the presence of hyperphosphorylated tau protein in the hippocampal formation from AD patients, and to explore the possible correlation between PHF_Tau_ and AD stages (Augustinack et al., 2002), two isoforms of PHF_Tau_ were quantified: PHF_Tau-AT8_ and PHF_Tau-pS396_.

In all hippocampal regions analyzed, we observed a higher density of PHF_Tau-pS396-ir_ (neurons per volume) than PHF_Tau-AT8-ir_, as previously observed in the CA1 region from AD patients particularly at the advanced stages of the disease (Su et al., 1994, 1996; Kimura et al., 1996; Blazquez-Llorca et al., 2010; Furcila et al., 2018); Supplementary Table S1; Figure 2B). The highest neuronal densities of PHF_Tau-pS396-ir_ and PHF_Tau-AT8-ir_ were observed in CA1, followed by the subiculum, whereas CA3 and DG presented lower values (Supplementary Table S1; Figure 2B). In addition, PHF_Tau-pS396-ir_ neuronal density was more variable between patients than PHF_Tau-AT8-ir_ neuronal density, particularly in the CA1 region. Moreover, the density of PHF_Tau-pS396-ir_ in CA1 was very close to the estimated overall neuronal densities (estimated in Nissl-stained and NeuN_-ir_ sections), suggesting that the majority of neurons in CA1 from AD patients contain PHF_Tau-pS396_. A ratio between the number of PHF_Tau-ir_ neurons and the total neurons estimated by Nissl-staining was calculated in CA3, CA1, and subiculum. These ratios showed that in CA3, PHF_Tau-AT8-ir_ neurons represent about 8% of the total neurons, and 10% in the subiculum, whereas PHF_Tau-pS396,_ was present in 13% of the CA3 neurons, and in 32% of the subicular neurons. In CA1, PHF_Tau-AT8-ir_ neurons represent about 31% of the neurons, while this figure reached 146% in the case of PHF_Tau-pS396_, that is, in CA1 the number of PHF_Tau-pS396-ir_ neurons exceeded the total number of total Nissl-stained neurons by 46% (Figure 2C; Supplementary Table S2), which could be explained by the labeling of extracellular NFTs or “ghost tangles” (see “Discussion” section).

Correlation analysis of the relative percentage of PHF_Tau-pS396_ neurons and the total neuronal population (estimated by Nissl-staining) showed a significantly negative correlation (*P* = 0.0037; *r* = −0.791; Spearman).

### Analyses of Aβ_-ir_ Plaques

**Figure 4:**
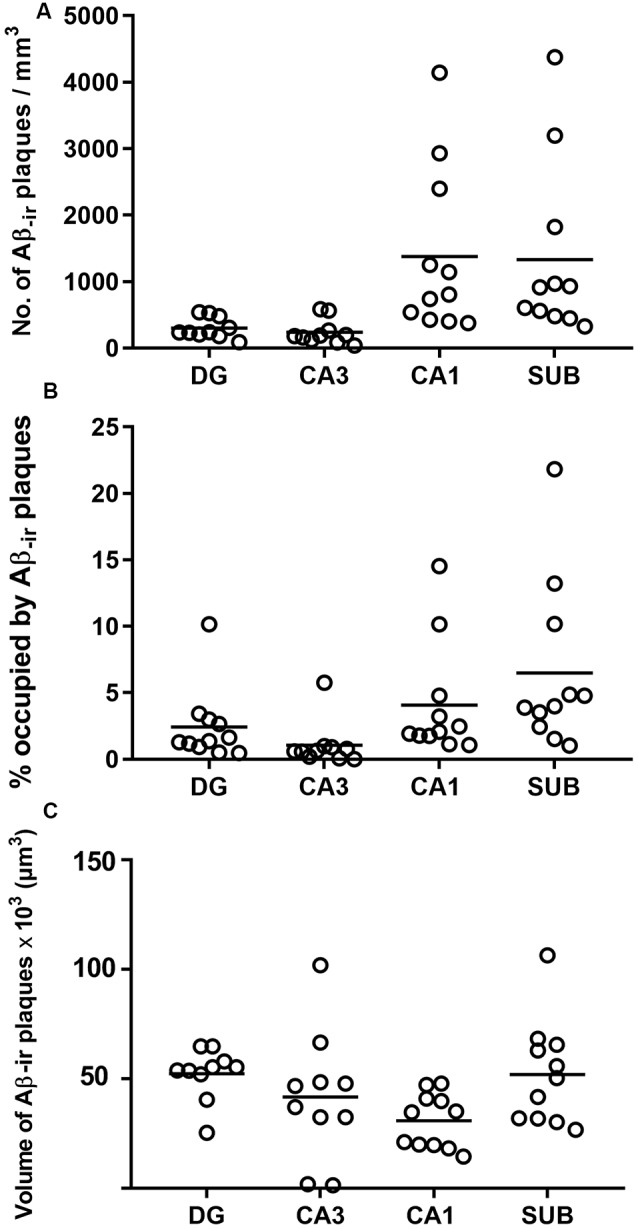
Plots to illustrate the estimated features of the Aβ_-ir_ plaques in the examined hippocampal fields. Number of Aβ_-ir_ plaques per volume **(A)**, the volume occupied by Aβ_-ir_ plaques **(B)**, and the estimation of Aβ_-ir_ plaque average volume **(C)**. DG, dentate gyrus; CA1–CA3, *cornu ammonis* fields; SUB, subiculum.

Estimation of the number of Aβ_-ir_ plaques per volume showed that CA1 presented the highest values, followed by the subiculum, DG and CA3 (Supplementary Table S1; Figure 4A). In addition, CA1 and subiculum not only presented the highest densities of plaques per volume (*p* < 0.0001; KW: 22.33), but they also displayed a higher proportion of tissue volume occupied by plaques (*p* = 0.0002; KW: 19.21; Supplementary Table S1; Figure 4B). By contrast, we found that the average volume of Aβ_-ir_ plaques (Supplementary Table S1; Figure 4C) was significantly higher (*p* = 0.0198; KW: 9.86) in the DG (52,350 μm^3^).

In summary, DG and CA3 displayed the lowest number of plaques per volume, and although plaques in these regions were bigger, the relative volume they occupied in these regions was lower. By contrast, CA1 and subiculum presented a higher number of Aβ_-ir_ plaques per volume and also showed higher proportions of tissue occupied by plaques, although the plaque size was relatively small.

## Discussion

In the current study, we used coronal sections of the human hippocampus at the level of the hippocampal body from patients with AD to estimate the density of neurons, NFTs and amyloid plaques per mm^3^ in different hippocampal fields. The present study is based on the analysis of relatively few sections of the hippocampus from these patients; therefore, the estimation should not be extrapolated to the whole hippocampus. We found that hippocampal CA1 field was the region most affected by the presence of numerous PHF_Tau-ir_ elements and Aβ-ir plaques, compared to other hippocampal fields of the same patients. The subiculum showed moderate levels of PHF_Tau-ir_ neurons and Aβ-ir plaques, while DG and CA3 were less affected by the presence of these pathological elements.

### Neuronal Densities

No significant differences in neuronal densities were found between the CA1, CA3 and subiculum within a given patient or between these hippocampal fields from different patients, except in AD patients with HS. A very low neuronal density was found in CA1 in the HS patients, with the density being significantly lower compared to CA3 and subiculum of the same patients as well as compared to CA1, CA3 and subiculum of the patients without HS. We examined 11 AD cases (age range: 69–89 years old) and the neuronal density in CA1 ranged from 2,475 to 8,458 neurons/mm^3^ in those cases with HS, and from 8,430 to 15,187 neurons/mm^3^ in the rest of the AD patients. Although age-matched controls for comparison with the AD cases would clearly be ideal, it should be noted that the neuronal density range of the AD patients without HS was similar to the control group described in a previous study performed in our laboratory using the same methods of quantification, despite the age differences (Andrioli et al., 2007). In this Andrioli et al.’s (2007) study, three autopsy control cases were examined and the neuronal densities ranged from 10,744 to 14,024 neurons/mm^3^ (age range: 49–69 years old). Moreover, the CA3 and subiculum from AD patients displayed neuronal densities (8,936 in CA3; 9,503 in subiculum) that were similar to those found in these control cases (9,852 ± 978 in CA3; 10,186 ± 2,672 in subiculum). Furthermore, in the same study by Andrioli et al. (2007), the neuronal densities in the control CA1 region obtained from biopsies taken from epileptic patients (age range: 21–65 years old) were estimated to range from 11,057 to 13,194 neurons/mm^3^. Therefore, although our previous studies were based on a relatively small number of cases, they did suggest that the density of neurons in the control CA1 is similar in individuals with ages ranging between 21 and 69 years old. Indeed, a rather small change (10%) in the number of neocortical neurons was found as a function of age by Pakkenberg and Gundersen (1997) in a study which covered ages ranging from 20 to 90 years old. However, since the AD patients show brain atrophy (e.g., van de Pol et al., 2006; although we did not measure the total volume of the hippocampus), it is obvious that there is a decrease in the absolute number of neurons in AD patients despite the fact that that the number of neurons per mm^3^ was similar to that found in previously published results in healthy individuals.

The vulnerability of CA1 to neurodegeneration has been widely investigated (reviewed in Duvernoy, 2005) and it has been reported that this particular region is very susceptible to metabolic changes (reviewed in Bartsch and Wulff, 2015). The greatest reduction in neuronal density was observed in those AD cases which also showed HS (Table 1, Supplementary Table S1; Figure 2A), while non-sclerotic AD patients displayed higher neuronal densities in the CA1 region, with values similar to control cases (see “Results” section). Since the majority of neurons in CA1 are pyramidal cells and severe neuronal loss was found in CA1 from AD patients with HS, it follows that their projection sites lack their normal inputs from CA1. However, how and when neuronal loss—related to AD progression—impacts on the hippocampal connectivity and function remains to be elucidated.

Remarkably, in CA1, we found that the density of PHF_Tau-pS396-ir_ neurons reaches the total population of NeuN_-ir_ neurons, and even surpasses the neuronal densities estimated in Nissl-stained sections. That is, virtually all CA1 neurons from AD patients were labeled by the anti-PHF_Tau-pS396_ antibody. Thus, some of the PHF_Tau-pS396-ir_ elements that surpass the total neuronal population may correspond to extracellular labeled NFTs. In the estimations performed in Nissl-stained sections, a neuron was quantified only if its nucleus was visible; neurons that had lost their nucleus or cellular integrity were not counted. It has been reported that in AD patients there is a pathological degeneration of PHF_Tau-ir_ neurons, which leads to a loss of the neuronal integrity, resulting in an extracellular NFT—the so-called “ghost tangle” (Braak et al., 1994). These ghost tangles have been seen in late stages of AD and they are phosphorylated, mainly at Ser396 (Kimura et al., 1996). In the present study, we found that cases with low neuronal density displayed more PHF_Tau-pS396_ extracellular tangles, suggesting that neuronal loss has occurred to a great extent due to AD-related tangle pathology. This finding may provide a morphological correlate of severe cell loss in AD, which has been referred to as hippocampal sclerosis in the literature (Dickson et al., 1994; Velez-Pardo et al., 2004; Attems and Jellinger, 2006; Amador-Ortiz et al., 2007).

Thus, in CA1 from AD patients, some of the neurons might have lost their integrity, functionality and normal connectivity. Indeed, a loss of dendritic spines and dendritic atrophy in pyramidal neurons containing well-developed NFTs has been described in AD patients (Merino-Serrais et al., 2013). So, progressive changes in the PHF_Tau-pS396-ir_ neurons may develop into morphological and functional alterations, and, finally, lead to a total degeneration of the neuron resulting in extracellular NFT. Moreover, it has been demonstrated that CA1 neurons show a heterogeneous expression pattern of PHF_Tau_ proteins. For example, in a previous study performed in our laboratory in CA1 regarding the co-expression of PHF_Tau-pS396_ and PHF_Tau-AT8_ in the same patients as in the present work, we found that 64% of the labeled neurons expressed only PHF_Tau-pS396_, 28% showed both markers and 8% displayed only PHF_TauAT8-ir_ (Furcila et al., 2018). Some studies have proposed that PHF_Tau-AT8_ is a specific marker of early stages, while PHF_Tau-pS396_ appears in late stages (Su et al., 1994, 1996; Regalado-Reyes et al., 2019). Nevertheless, other authors have suggested that PHF_Tau-pS396_ occurs in the early stages and has a dynamic pattern of expression throughout the course of the disease, while PHF_Tau-AT8_ appears in advanced stages (Mondragón-Rodríguez et al., 2014). Our results are in line with the latter study, since we found higher densities of PHF_Tau-pS396-ir_ neurons than PHF_Tau-AT8-ir_ neurons in all examined AD patients (which corresponded to the IV-V stages on the Braak scale). The role of intracellular PHF_Tau_ is an open debate; it has been suggested that its presence does not necessarily indicate severe and irreversible damage (Gong and Iqbal, 2008; Avila et al., 2012; Merino-Serrais et al., 2013; Polanco et al., 2017) and intracellular PHF_Tau_ has been reported in CA1 neurons in non-demented elderly patients (Ferrer, 2012). However, since PHF_Tau_ may impair the expression of another protein or proteins, and may affect the course of the disease, it is difficult to establish a hyperphosphorylation stage-dependent pattern.

### Aβ Plaques Density

CA1 also displayed the highest values for the density of Aβ_-ir_ plaques. Previous studies in our laboratory in the CA1 of AD patients have shown that the presence of Aβ_-ir_ plaques leads to local alterations, including changes in pyramidal cell morphology and innervation (e.g., Garcia-Marin et al., 2009; Blazquez-Llorca et al., 2010; Merino-Serrais et al., 2013; Antón-Fernández et al., 2017). Moreover, our previous study—on Aβ_-ir_ plaque distribution in CA1—revealed that most plaques were mainly located in the stratum pyramidale followed by the stratum radiatum, suggesting that the basal and apical dendritic arborization located in these CA1 layers might be severely affected in AD (Furcila et al., 2018).

Aβ_-ir_ plaques have been proposed to trigger glial activation (D’Andrea et al., 2001; Jung et al., 2015; for a review see Chun and Lee, 2018), which—in turn—has been proposed to be part of the inflammatory process associated with the presence of both PHF_Tau_ and Aβ in AD (Parbo et al., 2017). Microglial clustering has been reported in the vicinity of Aβ_-ir_ plaques in neocortical tissue from AD patients (Serrano-Pozo et al., 2013). In particular, activated microglia may internalize Aβ, contributing to the stabilization of plaque size (Rajendran et al., 2006; Serrano-Pozo et al., 2013; Asai et al., 2015), but the toxicity of pathological protein forms in late stages of AD may cause altered microglial functionalities. If microglial cells fail to clear soluble Aβ, they may dump it into the extracellular space again, making the formation of more Aβ_-ir_ plaques possible. In the present study, we found that regions with a larger number of Aβ_-ir_ plaques per volume (CA1 and subiculum) presented smaller plaques than those regions with less Aβ plaques (DG and CA3).

### Individual Variability

Our data were derived from relatively few AD demented patients (*n* = 11), precluding the extrapolation of the results to the whole population of patients with AD. Although examined patients were clinically rather homogeneous (Table 1), several characteristics should be considered regarding our current results. Our present data showed a remarkable variability in the neuronal density in the AD samples. Estimation of neuronal densities performed in Nissl-stained and NeuN-immunostained sections did not show differences in any of the analyzed regions. However, data derived from NeuN-immunostained sections showed greater variability, both between regions and patients. It has been reported that a delay in fixation of postmortem human brain tissue usually causes artifacts, such as disruptions in membrane continuity and an increase in the volume of the extracellular space, among other geometrical distortions (e.g., Tang et al., 2001). These features represent an important limitation when performing stereological counts, because the preservation of tissue volume is critical to obtain reliable estimates. Indeed, a recent study revealed that rat brain tissue undergoes significant metabolic changes that are accentuated with longer postmortem times and showed that this affects the visualization of certain antibodies, including anti-NeuN (Gonzalez-Riano et al., 2017). Quantitative studies on neuronal densities performed in human brain tissue with a large postmortem delay (up to 24 h) may provide data that differ from our results (Simic et al., 1997; Andrade-Moraes et al., 2013). While the preservation of human brain tissue with large postmortem delay might be acceptable for certain qualitative observations (e.g., Simic et al., 1997; Wittner et al., 2006; Andrade-Moraes et al., 2013), it might not be suitable for comparative studies performed in different laboratories. In addition, brain tissue fixing techniques may also alter stereological estimates, as studies have revealed a reduction in immunoreactivity using formalin (Lyck et al., 2009).

The source of variability may also be attributable to the individual cognitive reserve. Cognitive reserve has been proposed as a compensatory mechanism, which serves as protection against neuronal degeneration, allowing variable numbers of PHF_Tau-ir_ neurons and Aβ_-ir_ plaques to be presented (Tucker and Stern, 2011; Lazarczyk et al., 2012; Steffener and Stern, 2012; Stern, 2012; Avila et al., 2015; Hoenig et al., 2017).

Finally, Aβ_-ir_ plaques are observed in brain samples from non-demented cases, and clinical trials of AD therapies centered on the amyloid protein have failed to treat AD-associated cognitive impairment; thus, the amyloid pathology may not be solely responsible for the cognitive decline observed in AD (reviewed in Anderson et al., 2017; Morris et al., 2018). Moreover, in AD, the alterations are not only initiated by PHF_Tau_ and Aβ proteins, but probably also by the presence of other proteinopathies (Spires-Jones et al., 2017; Robinson et al., 2018). The interaction of altered proteins, and not their separate action, may modify the progression of AD. Thus, our study is in line with the idea that AD is not a unique entity but varies from one patient to another as a result of factors including—but not limited to—age, gender, co-pathologies and medical treatments.

In summary, we found that in AD patients CA1 showed the highest number of NFTs and Aβ plaques, whereas DG and CA3 displayed the lowest number of NFTs and Aβ plaques. Furthermore, AD patients have a variable neuronal loss in CA1 due to tangle-related cell death, which seems to correlate with the presence of extracellular tangles.

## Data Availability Statement

The datasets generated for this study are available on request to the corresponding author.

## Ethics Statement

The studies involving human participants were reviewed and approved by Banc de Teixits Neurologics from Hospital Universitari Clinic de Barcelona (Spain) and Banco de Tejidos Fundación CIEN (Madrid, Spain). The patients/participants provided their written informed consent to participate in this study.

## Author Contributions

JD and LA-N oversaw and designed the project. DF processed the tissue. DF and MD-Á performed data acquisition and initial analysis; they prepared the figures. LA-N supervised data analysis. DF and LA-N drafted the initial manuscript. All authors revised and contributed to the final version of the manuscript.

## Conflict of Interest

The authors declare that the research was conducted in the absence of any commercial or financial relationships that could be construed as a potential conflict of interest.

## Abbreviations

ABC: avidin-biotin complex
Aβ: amyloid-β
AD: Alzheimer’s disease
CA: *cornu ammonis* fields
DG: dentate gyrus
HS: hippocampal sclerosis
-ir: immunoreactive
MTL: medial temporal lobe
NFTs: neurofibrillary tangles
PHF_Tau_: paired helical filaments of tau protein
SD: standard deviation.
