# Supplementary material for: Subregional Density of Neurons, Neurofibrillary Tangles and Amyloid Plaques in the Hippocampus of Patients With Alzheimer’s Disease
Source: Front Neuroanat. 2019 Dec 19;13:99. doi: 10.3389/fnana.2019.00099 (PMC6930895; doi:10.3389/fnana.2019.00099)
Supplement: Supplementary file 1 [file Table_1.doc]

**Supplementary Table S1**. Data summary of stereological estimations in AD patients per region and labeling. DG: dentate gyrus; CA3−CA1: *cornu ammonis* field; SUB: subiculum. (-): Data not available. ^*^ indicates AD patients with hippocampal sclerosis.

| **Region** | **Patient** | **NeuN-ir per volume (mm^3^)** | **Nissl-stained per volume (mm^3^)** | **AT8-ir neurons per volume (mm^3^)** | **pS396-ir neurons per volume (mm^3^)** | **Aβ-ir plaques per volume (mm^3^)** | **Plaque volume (µm^3^)** | **% Occupied by Aβ-ir plaques** |
| --- | --- | --- | --- | --- | --- | --- | --- | --- |
| **DG** | Az1 | - | - | 759 | 137 | 182 | 53683 | 1.0 |
|  | Az2 | - | - | 666 | 571 | 178 | 57913 | 1.0 |
|  | Az3 | - | - | 407 | 174 | 232 | 53866 | 1.2 |
|  | Az4^*^ | - | - | 1957 | 391 | 410 | 55295 | 2.3 |
|  | Az5 | - | - | 82 | 118 | 156 | 25256 | 0.4 |
|  | Az6^*^ | - | - | 298 | 289 | 175 | 40394 | 0.7 |
|  | Az7^*^ | - | - | 922 | 424 | 67 | 52101 | 0.4 |
|  | Az8^*^ | - | - | 795 | 263 | 137 | 64788 | 0.9 |
|  | Az9^*^ | - | - | 600 | 477 | 365 | 55346 | 2.0 |
|  | Az10 | - | - | 1148 | 325 | 401 | 64860 | 2.6 |
| **Mean** |  |  |  | **763** | **317** | **230** | **52350** | **1.2** |
| **CA3** | Az1 | 10348 | 11574 | 818 | 199 | 149 | 32493 | 0.5 |
|  | Az2 | 8318 | 7873 | 729 | 669 | 65 | 66584 | 0.4 |
|  | Az3 | 9201 | 8347 | 875 | 444 | 122 | 48414 | 0.6 |
|  | Az4^*^ | 1706 | 6269 | 469 | 3193 | 445 | 1348 | 0.1 |
|  | Az5 | 7828 | 8342 | 357 | 98 | 139 | 32415 | 0.5 |
|  | Az6^*^ | 3029 | 9755 | 476 | 1059 | 31 | 46727 | 0.1 |
|  | Az7^*^ | 10219 | 10801 | 437 | 518 | 201 | 37036 | 0.7 |
|  | Az8^*^ | 10864 | 11550 | 1111 | 1503 | 94 | 1856 | 0.0 |
|  | Az9^*^ | 3263 | 9194 | 954 | 1964 | 145 | 47851 | 0.7 |
|  | Az10 | 7260 | 7224 | 659 | 1015 | 429 | 101974 | 4.4 |
| **Mean** |  | **7203** | **9093** | **689** | **1066** | **182** | **41670** | **0.8** |
| **CA1** | Az1 | 10167 | 8430 | 2423 | 3095 | 868 | 18136 | 2.0 |
|  | Az2 | 12766 | 9292 | 2110 | 15021 | 326 | 40975 | 1.0 |
|  | Az3 | 11148 | 8747 | 1394 | 2967 | 305 | 47767 | 1.0 |
|  | Az4^*^ | 2790 | 2475 | 2093 | 11163 | 1821 | 19935 | 4.0 |
|  | Az5 | 15604 | 15187 | 2697 | 3140 | 286 | 47242 | 1.0 |
|  | Az6^*^ | 6113 | 7098 | 2166 | 9883 | 410 | 21105 | 1.0 |
|  | Az7^*^ | 7813 | 8458 | 2046 | 6888 | 560 | 14435 | 1.0 |
|  | Az8^*^ | 6570 | 7754 | 3429 | 8394 | 612 | 39818 | 2.0 |
|  | Az9^*^ | 3179 | 5086 | 941 | 12777 | 948 | 19728 | 2.0 |
|  | Az10 | 14733 | 12467 | 2922 | 7393 | 3145 | 35063 | 11.0 |
|  | Az11^*^ | 6486 | 6065 | 2158 | 15785 | 2225 | 34650 | 8.0 |
| **Mean** |  | **8852** | **8278** | **2216** | **8773** | **1046** | **30805** | **3.1** |
| **SUB** | Az1 | 9007 | 7642 | 367 | 629 | 708 | 41637 | 3.0 |
|  | Az2 | 7323 | 11114 | 952 | 5053 | 341 | 106437 | 3.6 |
|  | Az3 | 10635 | 11134 | 768 | 3272 | 425 | 62992 | 2.7 |
|  | Az4^*^ | 2070 | 7260 | 449 | 2012 | 3321 | 30197 | 10.0 |
|  | Az5 | 10014 | 13119 | 748 | 788 | 366 | 31924 | 1.2 |
|  | Az6^*^ | 4505 | 8968 | 991 | 2252 | 461 | 65622 | 3.0 |
|  | Az7^*^ | 6997 | 4267 | 1367 | 3563 | 247 | 31864 | 0.8 |
|  | Az8^*^ | 9175 | 8559 | 621 | 1849 | 735 | 50304 | 3.7 |
|  | Az9^*^ | 1139 | 8795 | 665 | 1899 | 693 | 26736 | 1.9 |
|  | Az10 |  |  | 681 | 3784 | 1385 | 55789 | 7.7 |
|  | Az11^*^ | 18665 | 14174 | 1514 | 7643 | 2427 | 68270 | 16.6 |
| **Mean** |  | **7953** | **9503** | **829** | **2977** | **1010** | **51979** | **4.9** |

**Supplementary Table S2. Density of PHF_Tau-AT8-ir_ and PHF_Tau-pS396-ir_ neurons per volume, and estimated percentages (in parentheses) considering Nissl-stained neurons as the total neuronal population in all examined hippocampal areas. CA3−CA1: *cornu ammonis* field; SUB: subiculum. ^*^ indicates AD patients with hippocampal sclerosis.**

| **Region** | **AD Patient** | **PHF_Tau-AT8-ir_ neurons/mm^3^ (%PHF_Tau-AT8-ir_ / Nissl-stained neurons)** | **PHF_Tau-pS396-ir_ neurons/mm^3^ (% of PHF_Tau-pS396-ir_ / Nissl-stained neurons)** |
| --- | --- | --- | --- |
| **CA3** | Az1 | 818 (7%) | 199 (2%) |
|  | Az2 | 729 (9%) | 669 (8%) |
|  | Az3 | 875 (10%) | 444 (5%) |
|  | Az4* | 469 (7%) | 3193 (51%) |
|  | Az5 | 357 (4%) | 98 (1%) |
|  | Az6* | 476 (5%) | 1059 (11%) |
|  | Az7* | 437 (4%) | 518 (5%) |
|  | Az8* | 1111 (10%) | 1503 (13%) |
|  | Az9* | 954 (10%) | 1964 (21%) |
|  | Az10 | 659 (9%) | 1015 (14%) |
| **Mean** |  | **689 (8%)** | **1066 (13%)** |
| **CA1** | Az1 | 2423 (29%) | 3095 (37%) |
|  | Az2 | 2110 (23%) | 15021 (162%) |
|  | Az3 | 1394 (16%) | 2967 (34%) |
|  | Az4* | 2093 (85%) | 11163 (451%) |
|  | Az5 | 2697 (18%) | 3140 (21%) |
|  | Az6* | 2166 (31%) | 9883 (139%) |
|  | Az7* | 2046 (24%) | 6888 (81%) |
|  | Az8* | 3429 (44%) | 8394 (108%) |
|  | Az9* | 941 (19%) | 12777 (251%) |
|  | Az10 | 2922 (23%) | 7393 (59%) |
|  | Az11* | 2158 (36%) | 15785 (260%) |
| **Mean** |  | **2216 (31%)** | **8773 (146%)** |
| **SUB** | Az1 | 367 (5%) | 629 (8%) |
|  | Az2 | 952 (9%) | 5053 (45%) |
|  | Az3 | 768 (7%) | 3272 (29%) |
|  | Az4* | 449 (6%) | 2012 (28%) |
|  | Az5 | 748 (6%) | 788 (6%) |
|  | Az6* | 991 (11%) | 2252 (25%) |
|  | Az7* | 1367 (32%) | 3563 (84%) |
|  | Az8* | 621 (7%) | 1849 (22%) |
|  | Az9* | 665 (8%) | 1899 (22%) |
|  | Az11* | 1514 (11%) | 7643 (54%) |
| **Mean** |  | **829 (10%)** | **2977 (32%)** |
